# The Intersection of ChatGPT, Clinical Medicine, and Medical Education

**DOI:** 10.2196/47274

**Published:** 2023-11-21

**Authors:** Rebecca Shin-Yee Wong, Long Chiau Ming, Raja Affendi Raja Ali

**Affiliations:** 1 Department of Medical Education School of Medical and Life Sciences Sunway University Selangor Malaysia; 2 Faculty of Medicine, Nursing and Health Sciences SEGi University Petaling Jaya Malaysia; 3 School of Medical and Life Sciences Sunway University Selangor Malaysia; 4 GUT Research Group Faculty of Medicine Universiti Kebangsaan Malaysia Kuala Lumpur Malaysia

**Keywords:** ChatGPT, clinical research, large language model, artificial intelligence, ethical considerations, AI, OpenAI

## Abstract

As we progress deeper into the digital age, the robust development and application of advanced artificial intelligence (AI) technology, specifically generative language models like ChatGPT (OpenAI), have potential implications in all sectors including medicine. This viewpoint article aims to present the authors’ perspective on the integration of AI models such as ChatGPT in clinical medicine and medical education. The unprecedented capacity of ChatGPT to generate human-like responses, refined through Reinforcement Learning with Human Feedback, could significantly reshape the pedagogical methodologies within medical education. Through a comprehensive review and the authors’ personal experiences, this viewpoint article elucidates the pros, cons, and ethical considerations of using ChatGPT within clinical medicine and notably, its implications for medical education. This exploration is crucial in a transformative era where AI could potentially augment human capability in the process of knowledge creation and dissemination, potentially revolutionizing medical education and clinical practice. The importance of maintaining academic integrity and professional standards is highlighted. The relevance of establishing clear guidelines for the responsible and ethical use of AI technologies in clinical medicine and medical education is also emphasized.

## Introduction

Accelerated by advancement of computing technology, the use of artificial intelligence (AI) in clinical medicine has seen many remarkable breakthroughs from diagnosis and treatment to prediction of disease outcomes in recent years [1]. As new technological applications continue to emerge, ChatGPT, a generative language model launched by OpenAI in November 2022 has essentially has essentially revolutionized the IT world.. What makes ChatGPT a promising tool is the vast amounts of data used in its training and its ability to generate human-like conversations covering diverse topics.

Over the past few years, AI involving various techniques have gained significance in clinical medicine, whereas the use of chatbots has been documented in the published literature, even before the launch of ChatGPT. For example, one study reported the use of a chatbot in the diagnosis of mental health disorders [2]. In another study, Tudor et al [3] reported various applications of chatbots and conversational agents in health care, such as patient education and health care service support. Many of these applications can be delivered via smartphone apps [3].

The use of AI in medicine, including the use of generative language models, is often accompanied by challenges and contentions. Some common challenges include privacy, data security, algorithmic transparency and explainability, errors and liability, as well as regulatory issues associated with AI medicine [4]. Lately, the use of generative language models in scientific writing has also stirred up controversies in the academic and publishing communities. Some journals have declined ChatGPT as a coauthor, whereas others have happily accepted manuscripts authored by ChatGPT [5].

Currently, numerous reviews on the use of generative language model in the field of clinical medicine have been reported, but mainly in the context of academic writing [6] and medical education [7]. However, viewpoints on that relate the use of ChatGPT in clinical medicine, and its implications for medical education are lacking. The inexorable march of technological innovation, exemplified by AI applications in clinical medicine, presents revolutionary changes in how we approach medical education. With the advent of AI platforms like ChatGPT, the landscape of pedagogical methodologies within medical education is poised for unprecedented change. This model's vast training on an array of data and ability to generate human-like conversations is particularly compelling.

Despite earlier uses of AI and chatbots in clinical medicine, the introduction of highly advanced models such as ChatGPT necessitates a rigorous examination of their potential integration within medical education. Understanding the challenges that coincide with AI use, such as privacy, data security, and algorithmic transparency, is crucial for a comprehensive, informed, and ethically grounded exploration of AI in medical education. Hence, this article aims to provide a perspective on ChatGPT and generative language models in clinical medicine, addressing the opportunities, challenges, and ethical considerations inherent in their use, particularly their potential as transformative agents within medical education.

## Generative Language Models and ChatGPT

Generative language models such as ChatGPT are trained on a massive amount of text data to understand natural language and generate human-like responses to a wide range of questions and prompts (instructions). “GPT” stands for “Generative Pretrained Transformer.” ChatGPT is an enhanced version of previous generations of GPTs (GPT-1, -2, -3, and -3.5) and a sibling model to InstructGPT (OpenAI). It is an AI-based language model designed to generate high-quality texts resembling human conversations [8]. The technology underpinning ChatGPT is known as transformer-based architecture, a deep machine learning model that uses self-attention mechanisms for natural language processing. The model was first introduced by a team at Google Brain in 2017 [9]. Transformer-based architecture allows ChatGPT to break down a sentence or passage into smaller fragments referred to as “tokens.” Relationships among the tokens are then analyzed and used for new text generation in a similar context and style as the original text.

A detailed discussion of the technology used in ChatGPT is beyond the scope of this viewpoint article. Briefly, ChatGPT is a fine-tuned model belonging to the GPT 3.5 series. Compared to earlier versions of GPT, some strengths of ChatGPT include its ability to admit errors, ask follow-up questions, question incorrect assumptions, or even decline requests that are inappropriate. There are 3 main steps in the training of ChatGPT. The first step involves sampling of a prompt (message or instruction) from the prompt library and collection of human responses. The data are then used in fine-tuning the pretrained large language model (LLM). In the second step, multiple responses are generated by the LLM following prompt sampling. The responses are then manually ranked and are used in training a reward model to fit human preferences. In the last step, further training of the LLM is achieved by reinforcement learning algorithms based on supervised fine tuning and reward model training in the previous steps [8].

Currently, the research preview version of ChatGPT is available to the public at no cost. Although ChatGPT is helpful in data sourcing, and some users speculate that ChatGPT will replace search engines like Google, it is noteworthy that several key differences exist between a chatbot and a search engine [10]. Table 1 summarizes the differences between a chatbot and a search engine.

**Table 1:** Differences between a chatbot and a search engine.

	Chatbot	Search engine
Purpose	To generate natural language text responses	To index and retrieve information from the internet
Input	Questions and queries raised by users	Keywords entered by users
Output	Natural language text in the form of human-like conversations	List of links to web pages and relevant information
Output	Responses generated are personalized and conversational	Retrieved information is factual and objective
Information type	In the form of conversational text	Web-based contents in the form of text, images, and videos
Technology	Transformer-based neural network architecture	A combination of technologies (eg, machine learning, natural language processing, and web indexing).

## Opportunities for Using Generative Language Models

Studies have reported the use of ChatGPT in several medical education–related areas. In one study, ChatGPT passed the United States Medical Licensing Examination (USMLE) [11] and in another, it outperformed InstructGPT in the USMLE, achieving a passing score equivalent for a year 3 medical student [12]. Fijačko et al [13] reported that ChatGPT generated accurate answers and provided logical explanations to Basic Life Support and Advanced Cardiovascular Life Support examination questions but was unable to achieve the passing threshold for both examinations. Savage [14] described the potential use of ChatGPT in drug discovery.

It is worth mentioning that researchers have explored the use of generative language models in health care prior to the launch of ChatGPT. For example, a generative language model has been used in COVID-19 public health response [15], explanation of treatment process to stakeholders [16], patient self-management [17], mental health screening [18], research participant recruitment [19], research data collection [20].

At present, the ability of ChatGPT’s to perform complex tasks required of clinical medicine awaits further exploration [21]. It has been shown that the performance of ChatGPT decreases with increased complexity of the task. For example, Mehnen et al [22] reported that the diagnostic accuracy of ChatGPT decreased with rare diseases when compared to that with common diseases. Despite current limitations, a growing body of research suggests that ChatGPT and other chatbots can be trained to generate logical and informational context in medicine. Some potential applications of ChatGPT in clinical medicine and medical education are summarized in Table 2 [12,19,23-28].

**Table 2:** Potential applications of ChatGPT in clinical medicine and medical education.

Area of research	Potential applications	Example	Study (year)
Learning in medical education	ChatGPT as a source of medical knowledge	ChatGPT could pass the USMLE^a^, showing its ability in generating accurate answers	Mbakwe et al [11] (2023)
Patient engagement and education	Provide information to patients, caretakers, and the public	Use of chatbots in prostate cancer education	Görtz et al [23] (2023)
Disease prevention	Provide counseling and gather information (eg, risk factors) for health screening	Use of chatbots in symptom screening for patients with autoinflammatory diseases, with high patient acceptability	Tan et al [24] (2023)
Participant recruitment	Analyze information from potential participants through conversations and medical records and streamlined information gathered	Comparing recruitment of research participants using chatbot versus telephone outreach	Kim et al [19] (2021)
Data collection	Review large volumes of data through conversations and medical records, use data collected (eg, medical history, investigation findings, and treatment outcomes) for pattern recognition in diseases, and correlate data (eg, demographics and risk factors) with diseases	Use of a chatbot (Dokbot) for health data collection among older patients	Wilczewski et al [25] (2023)
Clinical decision support and patient management	Review data on medical history, investigation findings, etc, and provide treatment recommendations, and support clinical decision-making by providing supplemental information	Application of ChatGPT in making diagnoses and patient management using clinical vignettes	Rao et al [26] (2023)
Drug discovery and development	Review large volumes of scientific data on drugs and identify gaps and potential targets	Use of pretrained biochemical language models for targeted drug design	Uludoğan et al [27] (2022)
Medical writing	Assist in medical writing and publication	Application of ChatGPT in case report writing	Hedge et al [28] (2023)

^a^USMLE: United States Medical Licensing Examination.

## Drawbacks of Using Generative Language Model

Information accuracy and authenticity are a great challenge for using chatbots. In one study, researchers asked ChatGPT to generate 50 abstracts from selected medical publications. The study reported that ChatGPT could generate convincing abstracts that escaped plagiarism detection. Further analysis showed that scientists had difficulties in differentiating the fabricated abstracts from the original ones [29]. In another instance, researchers asked the researchers observed that the ChatGPT produced nonexistent or erroneous references [30]. From these examples, it is worrisome to learn that chatbots can generate fabricated and incorrect information, or what is known as “artificial hallucination.” These “hallucinations” have significant implications, especially when it comes to life-and-death matters in the clinical setting.

Based on its performance in a parasitology examination, a Korean study reported that ChatGPT showed lower knowledge and interpretation ability when compared to medical students [31]. Therefore, ChatGPT may need further training and enhancement on its ability to interpret medical information. In addition, the uncertainty on how ChatGPT and other AI applications derive their information and the black box problem have always been a big challenge in AI medicine [32]. This further raises concerns of transparency and trust, which are 2 crucial elements in medicine.

The training period of ChatGPT was between 2020 and 2021. As of this writing, ChatGPT was unable to provide information beyond the training period. For example, based on the authors’ experience, ChatGPT failed to describe the Turkey-Syria earthquakes that took place in February 2023. This implies that further training is necessary for ChatGPT to provide up-to-date information, whereas training a large-scale AI model like ChatGPT is expensive and time-consuming. Moreover, it involves feeding ChatGPT with high volumes of information, which requires highly skilled personnel.

## Ethical Considerations

The use of AI models like ChatGPT may give rise to social, ethical, and medico-legal issues. This section discusses these challenges and the potential pitfalls associated with the use of ChatGPT.

### Privacy, Confidentiality, and Informed Consent

Patient privacy and confidentiality, as well as data protection are common issues of debate in AI medicine [33]. Integration of existing health care systems and medical records with ChatGPT may lead to such issues. Informed consent must be obtained from the patients before ChatGPT accesses their data. The requirements of informed consent may vary depending on the situations. Some additional elements may need to be included when obtaining informed content for application of AI in medicine. Some examples include the disclosure of algorithmic decision support, a description of the input and output data, an explanation on the AI training, as well as the right of a second opinion by a human physician [34]. It is important that physicians ensure privacy and data security, as a breach of confidentiality may lead to a breach of trust, which can negatively impact the doctor-patient relationship.

### Accountability, Liability, and Biases

Accountability and liability are other ethical considerations. As some medical errors are life-threatening, physicians and researchers must ensure safety and accountability when using AI to support diagnosis, clinical decision-making, treatment recommendations, and disease predictions. Other ethical issues include biased and inaccurate data, leading to unfair and discriminatory results. Therefore, it is important to ensure that AI applications used in research and clinical medicine are trained on representative and diverse data sets to avoid such biases.

In the context of generative language models, bias may be viewed as systematic inaccurate representations, distortions or assumptions that favor certain groups or ideas, perpetuate stereotypes or any incorrect judgments made by the model based on previous training. Biases in generative language models can be introduced through various sources, such as the training data, algorithms, labeling and annotation, as well as product design decisions and policy decisions. On the other hand, different types of biases can occur, which include demographic, cultural, linguistic, and political biases [35].

Using LLMs like ChatGPT in clinical decision-making may lead to other unintended consequences such as malpractice and lawsuits. The use of traditional decision support tools like clinical practice guidelines allow physicians to assess the reliability of information according to the source and level of evidence. However, AI models like ChatGPT may generate biased and incorrect output with a lack of transparency in data sourcing. AI models may treat all sources of data equally and fail to differentiate the data based on evidence levels [36]. Depending on how the question is phrased, ChatGPT may provide different answers for the same question. Hence, the physicians should take these issues into consideration and use ChatGPT with caution in clinical decision-making.

### Regulation of the Use of AI in Medicine

With the emergence of social, ethical, and legal issues associated with applications of AI in health care, there is a need to impose regulatory measures and acts to address these issues. The regulation of AI medicine varies in different parts of the world. For example, in the United States, a regulatory framework and an action plan were published by the Food and Drug Administration in 2019 and 2021, respectively. In the United States, the responsibilities of AI lie with the specific federal agencies [37].

On the contrary, the European Commission proposed a robust legal framework (the AI Act) that regulates applications of AI in not only medicine but also other sectors. AI applications in medicine must meet the requirements of both the AI Act and the EU (European Union) Medical Device Regulation [38]. Some areas under such regulation include lifecycle regulation, transparency to users, and algorithmic bias [37]. The European Union also regulates the data generated by AI models via the GDPR (General Data Protection Regulation). Under the GDPR, solely automated decision-making and data processing are prohibited [39].

### Academic Dishonesty

**Figure 1:**
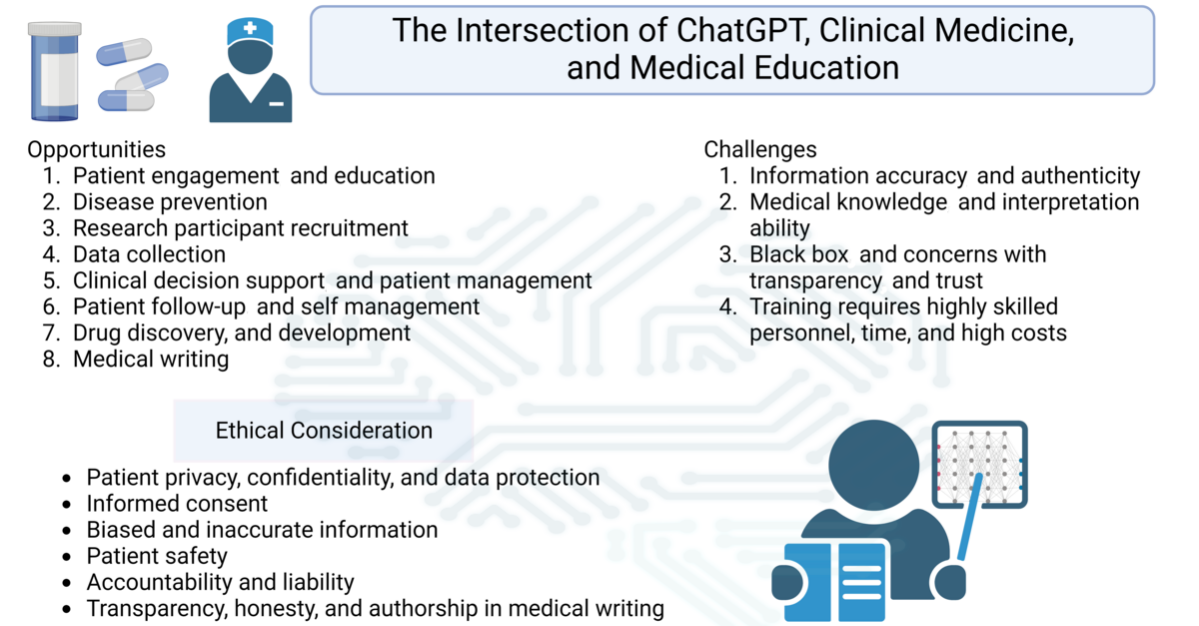
Overview of the use of ChatGPT in clinical medicine and medical education.

The use of ChatGPT in medical writing must be transparent, as it raises issues on academic dishonesty and fulfillment of authorship criteria, with some disapproving ChatGPT from being listed as an author in journal publications [5,40,41]. While the use of ChatGPT in clinical medicine and medical education allows easy access to a vast amount of information, it may raise issues like plagiarism and a lack of originality in scientific writing. Overreliance on ChatGPT may hinder the development of skills in original thinking and critical analysis. Figure 1 summarizes the use of ChatGPT in clinical medicine.

## Impact of Using AI Models in Clinical Medicine on Medical Training

As the use of AI models such as ChatGPT becomes more common in clinical medicine, it is likely to reshape the landscape of medical education and affect how medical students learn and handle information [42]. Some of the applications mentioned in Table 2 may also be applied in medical education. For instance, the use of ChatGPT in making diagnoses and patient management using clinical vignettes may enhance student learning experience and increase accessibility to learning resources [26]. The use of ChatGPT as a supportive tool in medical writing [28] may also have an impact on medical education. On the other hand, with the integration of AI models in medical education, medical educators will need to address certain issues such as accuracy and reliability of the information, as well as academic dishonesty.

Furthermore, while medical educators and physicians continue to explore the use of AI models in the clinical and research settings, there is an emerging need to introduce new elements in the teaching of medical ethics and medico-legal issues [43]. Whether medical educators readily embrace AI or approach it with caution, the growing presence of AI in our daily lives and the medical field cannot be denied. Therefore, it is time that medical educators re-evaluate the existing medical curriculum and incorporate these elements to prepare medical graduates for effective and ethical use of AI in their medical career.

## Conclusions

Generative language models have revolutionized the world. With its current state of technology, we believe that this new AI application has great potential in clinical medicine and medical education. “Garbage in, garbage out” is a common adage in computer science. Like any AI application, the key to the efficient use of ChatGPT depends on the quality of the training data. Given the fact that it can generate inaccurate and nonexistent information, generative language models still have room for improvement. Therefore, when using ChatGPT, physicians and medical students must always verify the information with reliable and evidence-based sources such as practice guidelines, peer-reviewed literature, and trusted medical databases.

While clinical researchers and physicians may use ChatGPT as a supportive tool, its role in replacing humans in complex data collection, analysis, and validation remains uncertain. Hence, the integration of AI in clinical medicine warrants further investigation. After all, when the chatbot makes mistakes, the ultimate responsibility lies with the human user. The use of generative language models in clinical medicine and medical education should also be ethical, taking into consideration patient safety, data protection, accountability, transparency, and academic honesty. When incorporating AI models in medical education, it is crucial that medical educators establish guidelines on the responsible and ethical use of applications such as ChatGPT. The importance of academic integrity, originality, and critical thinking should be emphasized to ensure that medical students uphold the highest professional standards throughout their medical education journey and their future clinical practice.

## Abbreviations

AI: artificial intelligence
EU: European Union
GDPR: General Data Protection Regulation
GPT: Generative Pretrained Transformer
LLM: large language model
USMLE: United States Medical Licensing Examination
